# HDAC3 role in medication consumption in medication overuse headache patients: a pilot study

**DOI:** 10.1186/s40246-015-0051-1

**Published:** 2015-11-05

**Authors:** Claudia Pisanu, Stefano Caproni, Donatella Congiu, Letizia M. Cupini, Alessio Squassina, George P. Patrinos, Ilenia Corbelli, Paolo Calabresi, Maria Del Zompo, Paola Sarchielli

**Affiliations:** Section of Neurosciences and Clinical Pharmacology, Department of Biomedical Sciences, University of Cagliari, SP8 Km. 0,700, 09042 Monserrato (CA), Italy; Neurologic Clinic, University of Perugia, Perugia, Italy; Headache and Cerebrovascular Diseases Center, Ospedale S. Eugenio, Rome, Italy; Department of Pharmacy, University of Patras, School of Health Sciences, Patras, Greece; IRCCS Fondazione “S. Lucia”, Rome, Italy; Institute of Neuroscience, CNR, Cagliari, Italy

**Keywords:** Medication overuse headache, HDAC3, Sequencing, Sodium valproate

## Abstract

**Background:**

Medication overuse headache (MOH) is a common and debilitating disorder characterized by generation, perpetuation, and persistence of intense chronic migraine, caused by overuse of analgesics, triptans, or other acute headache compounds. It has been suggested that MOH could share some pathogenetic mechanisms with other kinds of drug addiction. In this regard, histone deacetylases 3 (HDAC3) seems to have a role in the memory processes involved in extinction of drug-seeking behavior in animal models. HDAC3 is inhibited by sodium valproate, a drug with proven efficacy in MOH. Recent evidence suggests an involvement of genetic factors in predisposition to medication overuse.

**Results:**

In this association study, we sequenced all exons, intron/exon junctions, and 3′-5′UTR regions of *HDAC3* in 23 MOH patients to investigate its role in medication overuse. Associations between genotypes with continuous and dichotomous clinical characteristics were tested by multivariate analysis and Fisher’s exact test, respectively.

Sequencing of *HDAC3* revealed six single-nucleotide polymorphisms. The G allele of rs2530223 was significantly associated with the number of acute medications/month used and with the number of days/month in which medications were used (*p* = 0.006 and *p* = 0.007, respectively), but neither with headache frequency or intensity. None of the single-nucleotide polymorphisms (SNPs) was associated with clinical characteristics or response to sodium valproate.

**Conclusions:**

*HDAC3* could be implicated in excessive medication consumption in MOH patients. Our preliminary findings provide support for the need of further investigation on larger independent samples to confirm and extend the role of *HDAC3* in medication overuse headache.

## Background

Medication overuse headache (MOH) is a common and debilitating disorder characterized by generation, perpetuation, and persistence of intense chronic migraine, caused by overuse of analgesics, triptans, or other acute headache compounds [1]. Epidemiological studies show that MOH has become the third most frequent type of headache, with a prevalence of 1–2 % of the general population [2].

An increased risk of developing MOH has been shown in subjects with a family history of medication, drug, or alcohol abuse, suggesting a genetic predisposition to medication overuse [3]. Moreover, genetic variants have been suggested to have a role in migraine transformation into MOH [4], success of detoxification therapy [5], and relapse during the first year of follow-up [6].

The use of acute medication with psychotropic effects (barbiturates and opioids) has been associated with increased risk of migraine chronification [7]. MOH patients show symptoms of withdrawal [3], a high rate of relapses [8], and decision-making deficits [9].

A neuroimaging study with 18F-fluorodeoxyglucose positron-emission tomography in MOH patients showed that a number of dysmetabolic brain areas involved in pain-processing recovered after withdrawal of medication, while long-lasting dysfunction was observed in orbitofrontal cortex [10], an area suggested to be involved in drug dependence and addiction [11]. A recent study has also identified persistent dysfunctions in the mesocorticolimbic dopamine circuit in MOH patients [12]. On the basis of this evidence, it has been speculated that MOH shares some pathogenetic mechanisms with other kinds of drug addiction [13, 14].

Histone deacetylases 3 (HDAC3) is a protein expressed in almost all tissues, including brain [15], responsible for the deacetylation of lysine residues of the core histones. Among other functions, HDAC3 has been shown to be involved in negative regulation of long-term memory formation [16]. It has been suggested that molecular mechanisms underlying drug overuse are similar to those regulating long-term associative memory [17]. HDAC3 inhibition has been found to increase the memory processes involved in the extinction of drug-seeking behavior in animal models [18]. Moreover, HDAC3 expression has recently been found to be increased in rat dorsal striatal neurons which were activated during drug-seeking tests after prolonged methamphetamine withdrawal compared to nonactivated Fos-negative neurons [19].

Interestingly, this enzyme is inhibited by sodium valproate (VPA), an anticonvulsant and mood stabilizer approved by FDA for migraine prevention and shown to be effective in the treatment of chronic daily headache [20, 21]. Although VPA has been proven to be efficacious in the treatment of MOH with a history of migraine without aura, the exact mechanism of action of this drug in MOH is not known, and a large percentage of treated patients do not respond [22].

In this study, we carried out an explorative investigation on the role of *HDAC3* genetic variants in medication overuse and response to VPA treatment in a sample of patients with MOH by sequencing of the functional regions of the gene.

## Results

**Fig. 1:**
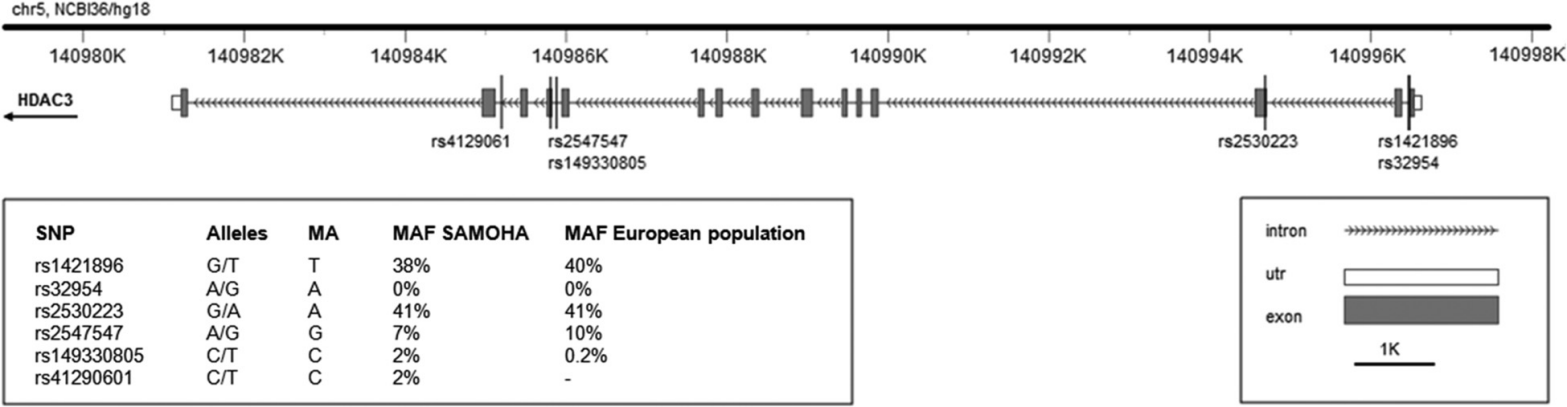
Gene diagram of *HDAC3* gene including SNPs identified in our sample. Abbreviations: *SNP* single-nucleotide polymorphism, *MA* minor allele, *MAF* minor allele frequency. Allele frequencies for European population are reported accordingly to 1000 Genome Project [29]; data obtained through VarioWatch [23]. The diagram was created with FancyGene [31]

Sequencing of the *HDAC3* gene revealed five intronic single-nucleotide polymorphisms (SNP) (rs1421896, rs32954, rs2547547, rs149330805, and rs41290601) and the synonym variant located in exon 3, rs2530223 (Fig. 1). Percentages of successfully genotyped samples were 90 % for rs1421896 and rs32954, and 100 % for all the others SNPs. Genotyping was successful in all individuals. Rs32954 had a minor allele frequency (MAF) lower than 0.01 and was excluded from the analyses. All SNPs were already present in public databases (UCSC Genome Bioinformatics; NCBI), and no new variants were identified in our patients. All SNPs were in Hardy-Weinberg equilibrium (HWE) in our sample.

Multivariate analysis showed that rs2530223 was significantly associated with the number of acute medications/month used and days/month in which acute medications were used (*p* = 0.02 and eta squared = 0.32 for both variables), but not with frequency or intensity of headache. Specifically, GA and GG carriers showed a higher medication abuse compared to AA carriers. Since the means of the associated variables were similar for GA and GG carriers, we hypothesized a recessive model for the effect of the A allele on medication consumption, and we used this model for subsequent analysis. When we grouped G allele carriers, we found higher significance for number of drugs/month abused and days/month of drug abuse for both variables (*p* = 0.006, eta squared = 0.31; *p* = 0.007, eta squared = 0.31, respectively; Table 1). G carriers were not different from other subjects in regard to age or MOH duration (Table 1). Although not significant, there was a sex difference in our groups (in G carriers female prevalence was 66 %, while it was 100 % among G noncarriers). For the numbers of drug/month abused, sex did not give a significant contribution to the model when tested as a covariate (*p* = 0.7). For the number of days/month in which medications were used, sex was significant as a covariate (*p* = 0.042), and the model including sex and rs2530223 was significantly associated with numbers of days/month of abuse (model: *p* = 0.003; rs2530223: *p* = 0.022; sex: *p* = 0.042). When correcting for headache frequency, the impact of rs2530223 on number of medications used and days of abuse remained significant (*p* = 0.03 for both variables).

**Table 1:** Association between clinical characteristics and genotypes of rs2530223

	AA	GA/GG	Statistics	*p* value	Eta squared
Women/men	5/0	12/6		0.27	
Age (years)	42.2 ± 10.9	43.1 ± 9.5	0.18	0.5	
MOH duration	3.9 ± 3.8	5.4 ± 3.6	0.81	0.37	
Number of headache days/month	18.6 ± 1.8	21.9 ± 4.1	3.04	0.09	0.12
Headache intensity	2.0 ± 0.7	2.2 ± 0.6	0.44	0.51	0.02
Number of drugs/month abused	14.2 ± 4.1	23.8 ± 6.7	9.47	**0.006**	0.30
Days/month of drug abuse	11.4 ± 2.6	19.0 ± 5.3	9.06	**0.007**	0.31
VPA responders/nonresponders	4/1	9/9	–	0.33	

Continuous data are expressed in mean ± standard deviation; dichotomous data are expressed as frequencies

Headache intensity was measured with a 4-point scale with 0 = no pain, 1 = mild headache, 2 = moderate headache, 3 = severe headache. Headache attacks and drugs used during the baseline period were collected using a headache diary. Additional information on how these variables were collected is available in Sarchielli et al., 2014 [22]. Significant variables are indicated in bold

*MOH* medication overuse headache, *VPA* sodium valproate

No *HDAC3* SNP was associated with response to VPA in our sample (data not shown). If used as a covariate in the linear model including rs2530223 and numbers of days/month of abuse, VPA response gave a nonsignificant contribution (*p* = 0.8), while the contribution of the SNP was still significant (*p* = 0.01). Thus, a different response to VPA does not seem to be influencing the hypothesized association between rs2530223 and medication consumption.

The analysis conducted with VarioWatch [23] showed that rs2530223 was predicted to have a “high” functional impact on *HDAC3*. VarioWatch assigns high functional impact to variants falling into an exonic splicing enhancer (ESE) site and into the protein domain. F-SNP database [24] showed that two out of four tools (ESRSearch and PESX) predicted an effect of rs2530223 on splicing regulation.

## Discussion

Our exploratory study investigated for the first time the association between *HDAC3* variants, medication overuse, and other clinical variables in a sample of 23 MOH patients. To this purpose, we sequenced exons, intron/exon junctions, and 3′-5′UTR regions of the gene, and we identified six SNPs, all of which resulted cataloged in public databases. Our analyses showed a significant association of the G allele of rs2530223 with higher medication consumption, but not with attack frequency or intensity of headache. A recent study conducted in a small sample of MOH patients showed that the amount of acute medications taken for month was able to predict the prognosis at a 1-year follow-up [25]. This observation was confirmed by a recent prospective study conducted in a larger sample of MOH patients showing that overuse severity was associated with negative outcome at a 3-year follow-up [26]. Thus, understanding the basis of medication consumption in MOH patients appears to be of great relevance.

Our preliminary results suggest that rs2530223 could be implicated in excessive medication consumption independently of the necessity of acute medication for pain relief. The GG genotype of the same SNP has been associated with cigarette smoking in a Korean population of schizophrenia patients [*p* = 0.0054; odds ratio (95 % confidence interval) = 4.33 (1.41-13.25)] [27]. Prevalence of smoking in schizophrenia patients is three times higher compared to general population, but carriers of the rs2530223 GG genotype seem particularly predisposed to develop this habit [27].

Although rs2530223 is a synonym variant, it could be in strong linkage disequilibrium with rare functional variants that our study was not powered enough to detect. Moreover, VarioWatch predicted a high risk of functional impact of this SNP on *HDAC3*. Our results support previous evidence suggesting an involvement of genetic factors in predisposition to medication overuse in MOH patients [28].

Allele frequencies of rs2530223 are population specific, as the MAF in subjects with European, West African, or East Asian ancestry is 0.4, 0.16, and 0.63, respectively, accordingly to data from the 1000 Genome Project [29] obtained through VarioWatch [23]. Further studies in other ethnic groups are needed to assess the potential association of this SNP with medication consumption in other populations.

In our sample, subjects with the rs2530223 AA genotype were characterized by a lower headache frequency (*p* = 0.09). Thus, an alternative explanation for our results would be that subjects with this genotype show lower medication consumption due to a lower number of days/month with headache. After correcting for headache frequency, the association between rs2530223 genotype and medication consumption was still significant (*p* = 0.03). While these results support our hypothesis that the SNP could play a role in medication overuse in these patients, the small sample size does not allow us to completely rule out a major contribution of headache frequency in driving significance. Findings from our study should be interpreted in light of the pilot nature of this investigation. The small sample size implemented could increase the risk for type 1 error and did not allow testing the effect of potentially relevant modifying factors in our model. Another limitation of the study is the small number of “AA” carriers in analysis under the recessive model. Strengths of our study include the prospective design and the choice to sequence *HDAC3* instead of target genotyping.

In conclusion, our pilot study suggests the involvement of *HDAC3* in excessive medication consumption in MOH patients. Our findings should be interpreted cautiously due to the limited sample size and might represent a first step of future extensive research aiming to clarify the role of *HDAC3* in medication overuse.

## Conclusions

*HDAC3* could be implicated in excessive medication consumption in MOH patients. These findings will serve as proof of concept for further investigations that will have to be performed on larger independent samples to confirm and extend our preliminary findings.

## Methods

### Subjects

This is an ancillary study from the sodium valproate in the treatment of medication overuse headache (SAMOHA) multicenter, randomized, double-blind, placebo-controlled study that enrolled 88 MOH patients for a 3-month treatment period with VPA (800 mg/day) or placebo after a 6-day outpatient detoxification regimen, followed by a 3-month follow-up. The 3-month responder rate (the proportion of patients achieving ≥50 % reduction in the number of days with headache per month) was higher in the VPA (45.0 %) than in the placebo arm (23.8 %) with an absolute difference of about 20 % (*p* = 0.043). VPA had safety and tolerability profiles comparable to placebo [22]. Informed written consent for the molecular study was obtained from 23 subjects included in the present work. All subjects undertook a venous blood draw before starting detoxification regimen and VPA therapy. The study was approved by the local Review Board. Table 1 shows the characteristics of the sample.

### Laboratory procedures

Genomic DNA was extracted from peripheral blood leukocytes according to classical salting-out protocol. The human *HDAC3* gene is located on chromosome 5q31 and comprises 15 exons, for a length of 16 kb. The sequenced regions included all exons, intron/exon junctions, and 3′-5′UTR regions.

Sequencing was carried out using the Big Dye Terminator sequencing kit on an ABI-PRISM 3130XL Genetic Analyzer (Applied Biosystems) according to the manufacturer’s protocol.

### Statistical analysis

Sequences were analyzed with Sequence Scanner software v1.0 and aligned to the reference sequence of *HDAC3* (UCSC Genome Bioinformatics) using BioEdit, version 7.2.5. Deviation from the HWE was tested with chi-square test. Polymorphisms with a MAF <0.01 were excluded from the analyses. Association between genotypes with continuous clinical characteristics and response to VPA was tested by multivariate analysis and Fisher’s exact test, respectively. Sex, headache frequency, and response to VPA were tested in the model as covariates. All the analyses were carried out with Haploview version 4.2 [30] and SPSS version 20 (IBM Statistics). A *P* value <0.05 was considered as statistically significant. Functional impact of associated variants was calculated with VarioWatch [23], a tool that assigns genomic variants a risk level on functional impact from “very low” to “very high”. Analysis was conducted using standard parameters (selected region: 50 kbp upstream and 50 kbp downstream of SNP position). Effect of associated variants on splicing regulation was assessed with F-SNP [24], a database that predicts SNPs effect on splicing regulation by integrating information of four different tools: ESEfinder, RescueESE, ESRSearch, and PESX.

## Abbreviations

ESE: exonic splicing enhancer
HDAC3: histone deacetylases 3
HWE: Hardy-Weinberg equilibrium
MAF: minor allele frequency
MOH: medication overuse headache
SAMOHA: sodium valproate in the treatment of medication overuse headache
SNP: single-nucleotide polymorphisms
VPA: sodium valproate
